# Neurocognitive impact of metal exposure and social stressors among schoolchildren in Taranto, Italy

**DOI:** 10.1186/s12940-019-0505-3

**Published:** 2019-07-19

**Authors:** Roberto G. Lucchini, Stefano Guazzetti, Stefano Renzetti, Michele Conversano, Giuseppa Cagna, Chiara Fedrighi, Augusto Giorgino, Marco Peli, Donatella Placidi, Silvia Zoni, Giovanni Forte, Costanza Majorani, Anna Pino, Oreste Senofonte, Francesco Petrucci, Alessandro Alimonti

**Affiliations:** 1 Icahn School of Medicine at Mount Sinai, New York, USA; 2 Department of Occupational Health, University of Brescia, Piazzale Spedali Civili, 1, 25123 Brescia, Italy; 3 Department of Public Health, Azienda USL – IRCCS, Reggio Emilia, Italy; 4 Department of Public Health, ASL, Taranto, Italy; 5 Department of Environment and Health, Italian National Institute of Health, Rome, Italy

**Keywords:** Metals, Neurotoxicity, Neurocognitive functions, Intellectual quotient, Social stressors, Schoolchildren

## Abstract

**Background:**

Metal exposure is a public health hazard due to neurocognitive effects starting in early life. Poor socio-economic status, adverse home and family environment can enhance the neurodevelopmental toxicity due to chemical exposure. Disadvantaged socio-economic conditions are generally higher in environmentally impacted areas although the combined effect of these two factors has not been sufficiently studied.

**Methods:**

The effect of co-exposure to neurotoxic metals including arsenic, cadmium, manganese, mercury, lead, selenium, and to socio-economic stressors was assessed in a group of 299 children aged 6–12 years, residing at incremental distance from industrial emissions in Taranto, Italy. Exposure was assessed with biological monitoring and the distance between the home address and the exposure point source. Children’s cognitive functions were examined using the Wechsler Intelligence Scale for Children (WISC) and the Cambridge Neuropsychological Test Automated Battery (CANTAB). Linear mixed models were chosen to assess the association between metal exposure, socio-economic status and neurocognitive outcomes.

**Results:**

Urinary arsenic, cadmium and hair manganese resulted inversely related to the distance from the industrial emission source (β − 0.04; 95% CI -0.06, − 0.01; β − 0.02; 95% CI -0.05, − 0.001; β − 0.02 95% CI -0.05, − 0.003) while the WISC intellectual quotient and its sub-scores (except processing speed index) showed a positive association with distance. Blood lead and urinary cadmium were negatively associated with the IQ total score and all sub-scores, although not reaching the significance level. Hair manganese and blood lead was positively associated with the CANTAB between errors of spatial working memory (β 2.2; 95% CI 0.3, 3.9) and the reaction time of stop signal task (β 0.05; 95% CI 0.02, 0.1) respectively. All the other CANTAB neurocognitive tests did not show to be significantly influenced by metal exposure. The highest socio-economic status showed about five points intellectual quotient more than the lowest level on average (β 4.8; 95% CI 0.3, 9.6); the interaction term between blood lead and the socio-economic status showed a significant negative impact of lead on working memory at the lowest socio-economic status level (β − 4.0; 95% CI -6.9, − 1.1).

**Conclusions:**

Metal exposure and the distance from industrial emission was associated with negative cognitive impacts in these children. Lead exposure had neurocognitive effect even at very low levels of blood lead concentration when socio-economic status is low, and this should further address the importance and prioritize preventive and regulatory interventions.

## Background

The role of metals as potential determinants of neurological diseases such as autism, attention deficit disorders and Alzheimer’s Dementia and Parkinson’s Disease is an emerging focus of human and public health research [1]. About 3% of developmental disabilities may be a consequence of environmental exposure to neurotoxicants and 25% of the interaction between environmental hazards and individual genetic predisposition [2, 3]. Extensive review of the current medical databases for chemicals that can be listed as developmental neurotoxicants [4], has shown about 100 molecules or elements, including six inorganic elements: aluminum (Al), manganese (Mn), arsenic (As), lead (Pb), mercury (Hg), and cadmium (Cd). In the priority list of the USA Agency for Toxic Substances and Disease Registry (ATSDR), As, Pb, Hg, Cd, are respectively ranked as first, second, third, and seventh, based on their frequency, toxicity and potential for human exposure [5]. All these metals can cross the placenta and the blood-brain barrier [6, 7], causing neurological impacts on general neurodevelopment [8–18], and more specifically on the Intellectual Quotient (IQ) [19], executive functions [20–22], memory [10, 21, 23], perceptual reasoning [23], and school performance [24, 25]. Although with a narrow homeostatic margin of safety [26, 27], selenium (Se) is both a potential neurotoxic element [27], and a chelating antioxidant that can eliminate neurotoxic metals through 26 Se-proteins including glutathione peroxidase [28].

Similarly to metals, socio-economic status (SES), home and family environments, psychosocial stressors including social violence act as developmental toxicant [29–31]. Social stressors impact neuroendocrine, immunological and autonomic nervous system and trigger dysfunctional and toxic behaviors within the family (such as inadequate parenting and smoking habits) [32]. While mechanisms involved in neurotoxicity of metals and social/maternal stressors are complex, epidemiological and biological evidence suggest both exposures may impact cellular response and oxidative damage [33, 34], with joint exposure especially targeting mitochondrial response and dysfunction [35].

The objective of this study was to assess the effect of co-exposure to As, Pb, Hg, Cd, Mn, Se and socio-economic stressors on the intellectual abilities including the IQ and its sub-scores and the executive functions among 6–12 years old children characterized by a variety of levels of exposure including subjects residing in the heavily polluted and socially disadvantaged areas of Taranto, in southern Italy.

## Material and methods

### Study area

Since 1964 the city of Taranto, in the Puglia region of southern Italy has been a highly industrial area that includes the largest iron- and steel-making operation in Europe and an oil refinery and a cement factory. The steel plant uses iron mineral, limestone and coal which are melted at temperatures over 1000 °C causing the iron compounds to release excess oxygen and turning into melted iron for steel production. This process causes airborne emission of metals, nitrogen oxides, sulphides, arsenide, carbon dioxide, organic compounds, and naturally occurring radionuclides, with potential contamination of water, soil and sediments. In 2002, the steel plant released the 30.6% of total dioxin emissions of Italy and in 2005 the estimated annual emission of Hg was over 2 tons [36]. Excess mortality, increased cancer incidence and hospital admission have been observed in this area compared to the local expected values [37], and biomonitoring measurements have shown increased levels of various toxic elements [38, 39].

### Sample selection and recruitment strategies

Five different sub-areas of Tamburi, Statte, Paolo VI, Taranto, Talsano, were identified at incremental distance from the industrial site, based on the average annual air monitoring data and particles deposition of urban pollutants measured in 2010 by the Environmental Agency (ARPA) of the Puglia Region [40]. Our power calculation estimated the total number of 300 children (aged 6–12, 50% males/females) were enrolled in the 12 primary schools located in the 5 sub-areas. Enrollment was obtained by informative meetings with children, their parents and teachers in each school and at a convenient schedule. Participation was voluntary and consented by the parents, as approved by the local Ethical Committee.

Overall 700 subjects were invited to participate to the study; 432 decided to adhere, yielding a participation rate of 62%, with no substantial differences among the study areas. Out of the 432 subjects 133 were excluded either because they did not meet the inclusion criteria of being born and raised in the target study areas, and having carried the entire pregnancy period of the mother in the same area at the time of recruitment or because of the exclusion criteria including familiarity of neurodegenerative diseases, diagnosis/treatments for neurological and psychiatric illnesses, hepatic or biliary diseases, dysmetabolic diseases, endocrine disorders, kidney disorders, previous total parenteral nutrition and uncorrected visual defects. In particular 21 subjects were excluded because they resulted as non-residents in the target areas, 16 because affected by clinical conditions, 2 for disagreement between the parents, 23 for refusal to participate after being enrolled, and 78 were recruited but not examined because the target population size of 300 subjects based on budgetary limitation was already reached, resulting in a final group of 299 participants.

### Biomonitoring of metal exposure

Whole blood, urine and hair were collected in the schools 1 day before the neuropsychological testing. Measures of Pb and Se in whole blood, As and Cd in urine, and Hg and Mn in hair were obtained with High Resolution-Inductively Coupled Plasma Mass Spectrometry (HR-ICP-MS) (ElementII, Thermo Scientific, Bremen, Germany). The analytical details are reported elsewhere [41, 42]. Hair Hg was determined with Direct Mercury Analyzer (DMA-80 Tricell, FKV, Bergamo, Italy) [43] and hair Mn with ICP-MS. For each matrix, blanks and appropriate certified reference materials were included for all analytical runs; all analytical methods were accredited by the Italian Accreditation Institute (ACCREDIA). Limit of detection (LOD) was determined applying the 3σ criteria.

### Sociodemographic and neuropsychological assessment

**Fig. 1:**
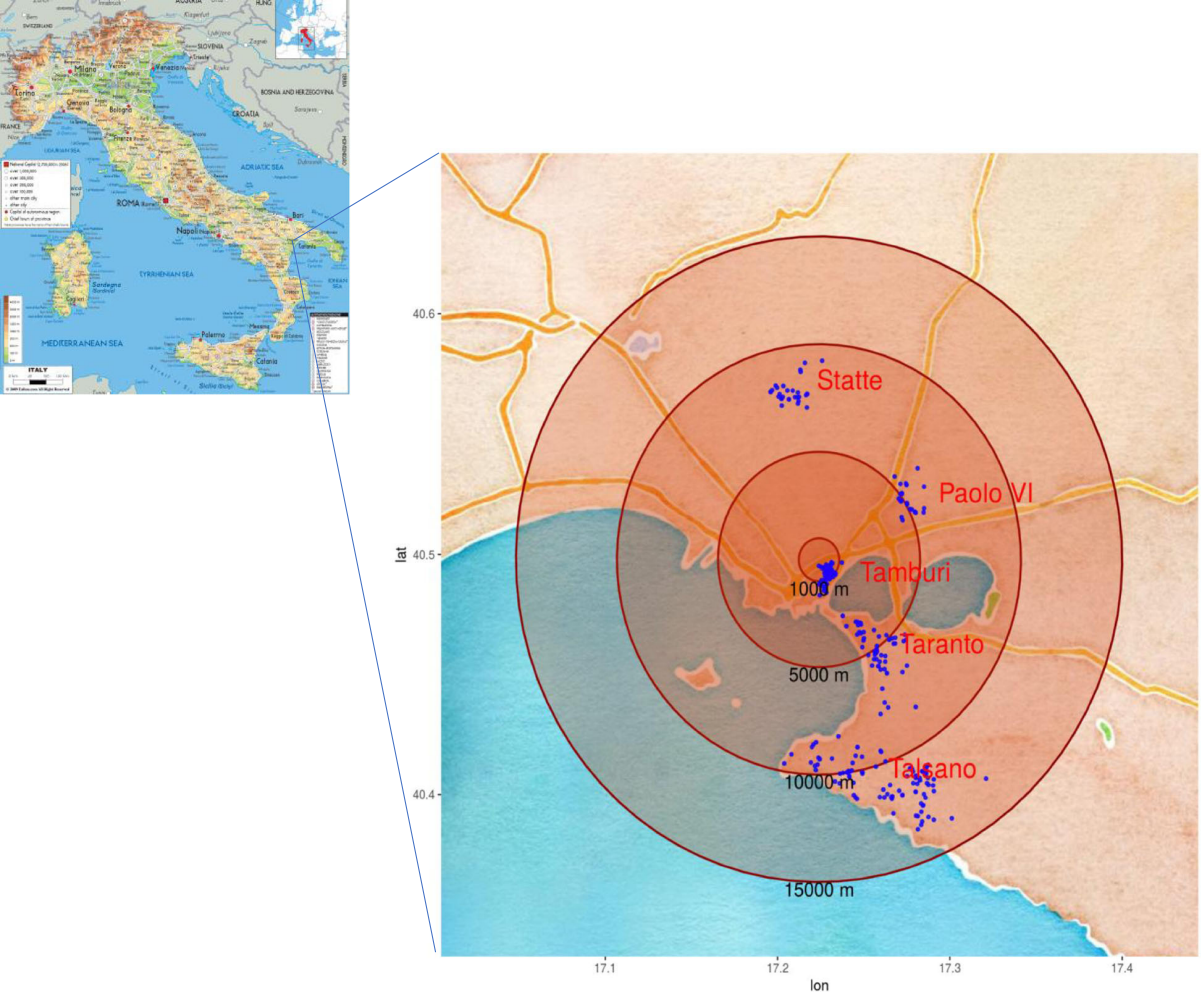
Distance from the point source. A map of the spatial distribution of the residence of the subjects enrolled in the study is shown, highlighting the distance from the source of the exposure. The localization of the city of Taranto is also provided

Quiet rooms were made available in each school for individual assessment of sociodemographic and lifestyle data through the administration of questionnaires to parents. The SES was calculated according to Cesana et al. [44] where parental education and occupation were classified based on age, level of education, occupational level and work-related stress perception, and then used to determine the three SES categories: low, medium and high. The distance from the point source was calculated for each participant as the distance between the home address and the closest point of the polygon delimiting the area of the steel plant (Fig. 1).

Information about the home environment, especially cognitive stimulation was collected through the Home Observation for Measurement of the Environment (HOME) questionnaire [45]. The neuropsychological assessment was conducted by four trained neuropsychologists who performed periodical inter-calibration of testing procedure and scoring. The testing protocol included the Italian version of the Wechsler Intelligence Scale for Children edition IV (WISC-IV) [46] (the Italian version of the most recent WISC-V is not yet available) for children’s cognitive assessment. The WISC-IV generates a full-scale IQ and also four composite standard scores: Verbal Comprehension Index (VCI), Visual-Perceptual Reasoning Index (PRI), Working Memory Index (WMI) and Processing Speed Index (PSI). These indices then can be grouped in two other scores: VCI and PRI as part of the General Ability Index (GAI) and WMI and PSI as part of the Cognitive Proficiency Index (CPI). The GAI index represents the estimate of general intellectual ability while the CPI is an estimate of the efficiency with which the information is processed in learning, problem solving and higher order reasoning. The executive functions were tested with the Rapid Visual Information Processing (RVP) to measure the sustained attention, Stop Signal Task (SST) for response inhibition, Spatial Working Memory (SWM) to provide measure of strategy and working memory errors and Stockings of Cambridge (SOC) to test spatial planning using problem-solving strategies, selected from the Cambridge Neuropsychological Test Automated Battery (CANTAB) [47]. The specific selected tests were the probability of hit and the total false alarms for the RVP, the between errors and the strategy for the SWM, the errors on stop and go trials, proportion of successful stops, mean correct reaction time, stop signal delay, stop signal reaction time and the total correct on stop and go trials for the SST and the problems solved in minimum moves for the SOC. The Raven’s progressive matrices (Standard Progressive Matrices, SPM) was utilized to assess the maternal non-verbal intellectual efficiency [48].

### Statistical analysis

Descriptive statistics were analyzed globally and stratified by categorized distance from the source of emission (0–1 km, 1–5 km, 5–10 km, 10–15 km). Median, 1st and 3rd quartiles were chosen as summary statistics for the biomarkers, as they exhibited strongly asymmetrical distributions. Preliminary comparison of the neurocognitive scores among the distance categories was performed with non-parametric Kruskal Wallis rank-sum test.

CANTAB RVP probability of hit was area hyperbolic sine transformed and SST mean correct reaction time on go trials and stop signal reaction time were log-transformed to gain a normal distribution. A logarithmic transformation was applied to the concentration of metal to allow linearization and mitigate the effect of extreme values. Biomarkers values were also standardized to have a better interpretability when the interaction term with the SES was applied. The relationship between exposure biomarkers, SES and neuropsychological outcomes was tested with mixed effects linear regression models to consider the nested structure of the data where the levels from the lower to the upper were class, school and area of residence. Bootstrap confidence intervals (CI) were used to test for significance of the parameters estimated in the models. To avoid a sample size reduction in multivariate analysis, we included the values <LOD in the dataset since they represent a proportion of observations lying within the distribution’s quantiles, and therefore can be considered informative.

The assessment of linearity between the independent variables and the outcomes was conducted preliminarily (not shown here) by using generalized additive models (GAMs) [49].

In addition to the effect of the exposure biomarkers and SES all regression models were adjusted for the covariates that we hypothesize to influence cognitive functions like sex, age, maternal non-verbal intelligence and cognitive stimulation besides the confounder distance from the point source. All statistical analyses, tables and graphs were performed with R 3.5.2 [50].

## Results

The socio-demographic data for the entire population which was also divided into four groups representing categorized increasing distance from the point source, are reported in Table 1.

**Table 1:** Socio-demographic characteristics

	Total *N* = 299	0–1 km *N* = 51	1–5 km *N* = 55	5–10 km *N* = 118	10–15 km *N* = 75
N (%)					
Sex					
Female	161 (53.8)	39 (76.5)	29 (52.7)	55 (46.6)	38 (50.7)
SES					
Low	124 (41.5)	35 (68.6)	26 (47.3)	38 (32.2)	25 (33.3)
Medium	103 (34.4)	15 (29.4)	19 (34.5)	39 (33.1)	30 (40.0)
High	72 (24.1)	1 (2.0)	10 (18.2)	41 (34.7)	20 (26.7)
Mean (SD)					
Age (yrs)	8.6 (1.5)	8.9 (1.6)	8.6 (1.4)	8.3 (1.4)	9.0 (1.5)
Maternal SPM	65.7 (23.8)	55.3 (24.9)	63.5 (8.1)	67.6 (21.7)	71.2 (20.9)
Distance (km)	6.3 (4.1)	0.7 (0.2)	2.7 (1.5)	7.2 (1.7)	11.5 (0.9)
HOME	5.5 (1.8)	4.7 (1.6)	5.6 (1.7)	5.8 (1.6)	5.8 (1.7)

Notes: Socio-demographic characteristics of the total population sample and divided by area of residence

*SPM* Standard Progressive Matrices, *HOME* Home Observation for Measurement of the Environment

Subjects were similarly distributed for age and sex, but not for SES, which was significantly lower in the areas closer to the emission sources (only 2%, Chi-squared *p*-value < 0.001) compared to those living at greater distance (34.7 and 26.7% of subjects with high SES at 5–10 km and 10–15 km respectively). Maternal SPM (Kruskall-Wallis rank sum test *p*-value = 0.005) and the HOME total score (Kruskall-Wallis rank sum test *p*-value < 0.001) showed a significant increase with the distance where the subjects at closer distance from the source had minor cognitive stimulation from the home environment. A much higher percentage of females characterized the subjects at closer distance while the proportion of gender at other distances was well balanced.

Descriptive statistics of biomonitoring data and their association with the distance from the source are shown in Table 2. Apart from BSe and HHg, all metal concentrations show a significant decrease at increasing distance, while BPb shows a non-significant decreasing trend. Table 3 presents the Spearman correlation matrix showing a moderate correlation among biomonitoring values: the highest correlation coefficient is between UAs and HHg of 0.276.

**Table 2:** Biomarkers summary statistics

	Min	Q1st	Median	Q3rd	Max	β coeff	95% CI
UAs (ng/mL)	1.1	5.1	8.3	15.1	797.3	−0.04	− 0.06, − 0.01
UCd (ng/mL)	0.0	0.3	0.4	0.7	1.8	−0.02	−0.05, − 0.001
HHg (ng/g)	21.2	271.1	476.6	744.7	4,357.0	0.04	0.02, 0.07
HMn (ng/g)	12.0	87.9	135.3	202.4	2,597.0	−0.02	−0.05, − 0.003
BPb (ng/mL)	2.6	6.4	8.4	11.0	35.9	−0.001	−0.01, 0.01
BSe (ng/mL)	82.2	126.8	142.7	161.0	263.0	0.01	0.001, 0.01

Notes: Biomarkers descriptive statistics and β coefficients and 95% confidence intervals (CI) of the association with the distance from the source (km)

*UAs* Urinary arsenic, *UCd* Urinary cadmium, *UHg* Urinary mercury, *HHg* Hair mercury, *BPb* Blood lead, *HMn* Hair manganese, *BSe* Blood selenium

**Table 3:** Spearman correlation among biomarkers

	UCd	UAs	BPb	BSe	HMn	HHg
UCd	1	< 0.001	0.418	0.006	0.262	0.021
UAs	0.253	1	0.099	0.061	0.058	< 0.001
BPb	−0.047	0.0953	1	0.320	0.334	0.549
BSe	−0.159	0.1081	0.058	1	0.426	0.003
HMn	0.065	0.109	0.056	−0.046	1	0.399
HHg	−0.133	0.276	−0.035	0.173	0.049	1

Notes: The lower triangle of the matrix shows the Spearman’s correlation coefficients; the upper triangle shows the *p*-values related to the null hypothesis ρ = 0

*UAs* Urinary arsenic, *UCd* Urinary cadmium, *UHg* Urinary mercury, *HHg* Hair mercury, *BPb* Blood lead, *HMn* Hair manganese, *BSe* Blood selenium

Table 4 shows the values of the IQ total score and sub-scores and the CANTAB tests in the total group and grouped by the categorized distance from the point source. The IQ total score and all the subtests, except PSI, showed significantly lower values at closer distance from the point source. Remarkably, the subjects residing at closer distance (0–1 km) from the emission sources showed a total IQ score of 15 points lower compared to those residing at greater distance (10–15 km).

**Table 4:** Neurocognitive outcomes summary statistics

	Total	0–1 km	1–5 km	5–10 km	10–15 km	*P*-value^†^
WISC-IV						
IQ	106.0 (94.0, 115.0)	94.0 (87.0, 107.0)	98.0 (90.5, 110.0)	108.0 (97.5, 118.0)	110.0 (99.5, 118.0)	< 0.001
GAI	108.0 (96.0, 116.0)	99.0 (88.0, 110.5)	102.0 (91.5, 114.0)	109.5 (101.0, 119.0)	112.0 (101.5, 122.5)	< 0.001
CPI	100.0 (91.0, 109.0)	94.0 (85.0, 102.5)	98.0 (87.0, 104.0)	104.0 (92.0, 111.0)	104.0 (94.0, 111.5)	< 0.001
WMI	100.0 (88.0, 109.0)	88.0 (82.0, 100.0)	94.0 (88.0, 100.0)	100.0 (94.0, 109.0)	106.0 (91.0, 109.0)	< 0.001
PSI	103.0 (94.0, 112.0)	103.0 (91.7, 111.2)	100.0 (91.0, 109.0)	103.0 (94.0, 112.0)	103.0 (93.2, 109.0)	0.379
PRI	106.0 (95.0, 117.0)	98.0 (90.5, 111.0)	99.0 (89.0, 111.0)	108.0 (100.0, 117.0)	109.5 (98.5, 119.0)	< 0.001
VCI	108.0 (98.0, 118.0)	98.0 (88.0, 108.5)	104.0 (92.0, 112.5)	110.0 (102.0, 118.0)	112.0 (105.5, 122.0)	< 0.001
CANTAB						
RVP						
Probability of hit	0.8 (0.7, 0.9)	0.750 (0.667, 0.896)	0.8 (0.6, 0.9)	0.8 (0.7, 0.9)	0.8 (0.7, 0.9)	0.170
Total false alarms	2.0 (1.0, 3.5)	2.0 (0.5, 3.0)	2.0 (1.0, 4.0)	2.0 (1.0, 3.0)	1.0 (1.0, 3.5)	0.055
SWM						
Between errors	54.0 (41.5, 63.0)	60.0 (43.0, 68.0)	56.0 (41.0, 64.0)	51.0 (39.0, 60.0)	54.0 (42.5, 61.5)	0.025
Strategy	38.0 (36.0, 40.0)	39.0 (37.0, 42.0)	38.0 (36.0, 40.0)	37.5 (35.0, 39.0)	38.0 (36.0, 40.5)	0.026
SST						
Direction errors on stop and go trials	3.0 (1.0, 7.0)	2.0 (0.5, 3.0)	3.0 (1.0, 6.5)	3.0 (1.0, 7.0)	3.0 (1.0, 8.5)	0.091
Proportion of successful stops	0.5 (0.4, 0.6)	0.5 (0.5, 0.6)	0.5 (0.4, 0.6)	0.5 (0.4, 0.6)	0.5 (0.4, 0.6)	0.662
Mean correct reaction time on go trials	722.0 (625.8, 878.4)	725.7 (655.6, 938.8)	703.8 (609.3, 884.9)	742.8 (633.6, 889.9)	691.5 (616.0, 803.7)	0.187
Stop signal delay	384.8 (286.5, 496.5)	404.4 (337.7, 478.8)	384.8 (271.7, 502.9)	398.1 (265.8, 526.7)	356.7 (283.4, 440.2)	0.219
Stop signal reaction time	282.1 (234.0, 344.8)	274.2 (236.8, 355.5)	285.8 (233.4, 341.8)	294.1 (240.6, 345.2)	271.5 (224.1, 334.4)	0.578
Total correct on stop and go trials	282.0 (275.0, 288.0)	283.0 (280.0, 289.0)	281.0 (275.0, 289.5)	282.0 (275.0, 289.0)	280.0 (273.0, 286.0)	0.188
SOC						
Problems solved in minimum moves	6.0 (5.0, 7.0)	7.0 (5.0, 8.0)	6.0 (5.0, 6.5)	6.0 (5.0, 7.0)	6.0 (5.0, 7.0)	0.071

Notes: Neurocognitive outcomes from the WISC-IV and CANTAB by distance from the source - Median (Q1, Q3)

*WISC-IV* Wechsler Intelligence Scale for Children edition IV, *IQ* Intellectual Quotient, *VCI* Verbal Comprehension Index, *PRI* Visual-Perceptual Reasoning Index, *WMI* Working Memory Index, *PSI* Processing Speed Index, *GAI* General Ability Index, *CPI* Cognitive Proficiency Index, *CANTAB* Cambridge Neuropsychological Test Automated Battery, *RVP* Rapid Visual Information Processing, *SST* Stop Signal Task, *SWM* Spatial Working Memory, *SOC* Stockings of Cambridge

†Kruskal-Wallis rank sum test

The CANTAB tests showed a similar trend, but only SWM between errors and strategy showed a statistically significant difference among the categorized distance (between errors: 60.0, 56.0, 51.0, 54.0 at 0–1, 1–5, 5–10 and 10–15 km respectively; strategy: 39.0, 38.0, 37.5, 38.0 at 0–1, 1–5, 5–10 and 10–15 km respectively).

We assessed the independent association of metal exposure and SES with the IQ total score and sub-scores and the CANTAB tests using a linear mixed model to consider the non-independence related to the school, the school class and the area of residence of each subjects, through nested random effects, adjusting for age, sex, maternal SPM, HOME total score, and distance from the point source (Table 5). None of the metals was individually associated with the IQ total and sub-scores, but considering the 95% CI of those parameters that showed a negative trend, BPb showed an upper limit close to zero in the association with IQ, GAI, CPI and VCI, similarly to UCd with IQ, GAI, CPI, WMI and VCI. The SES resulted associated with the IQ score, with highest levels showing IQ, GAI and WMI of about 5 points higher than the lowest SES level, and VCI around 7 points higher than the lowest SES level (IQ: β_high SES_ 4.8; GAI: β_high SES_ 5.6; WMI: β_high SES_ 5.3; VCI: β_high SES_ 7.1) which means a relative percentage change of about 6, 7 and 9% respectively, considering the ranges of the three cognitive outcomes ((64, 143), (61, 139), (64, 144) respectively).

**Table 5:** Linear mixed effect model testing metals and SES association with neurocognitive outcomes

	SES Mid vs Low	SES High vs Low	log (BPb)	log (HMn)	log (HHg)	log (UAs)	log (UCd)	log (BSe)
WISC IV								
IQ	1.3 (−2.3, 5.2)	4.8^a^ (0.3, 9.6)	−1.1 (− 2.7, 0.6)	0.2 (−1.6, 1.7)	−0.03 (− 1.9, 1.8)	0.9 (− 1.0, 2.7)	− 1.2 (− 3.0, 0.6)	0.1 (− 1.6, 1.9)
GAI	1.3 (− 2.5, 5.6)	5.6^a^(0.6, 11.1)	− 1.5 (− 3.3, 0.2)	1.1 (− 0.6, 2.9)	− 0.9 (− 2.9, 1.1)	1.7 (− 0.2, 3.6)	−1.2 (− 2.9, 0.7)	0.3 (− 1.5, 2.4)
CPI	0.6 (− 3.1, 4.5)	2.1 (− 2.8, 7.0)	− 1.2 (− 2.8, 0.5)	−1.1 (− 2.8, 0.6)	0.5 (− 1.2, 2.2)	−0.1 (− 2.0, 1.8)	−1.4 (− 3.1, 0.3)	−0.1 (− 1.8, 1.5)
WMI	1.9 (−2.1, 6.1)	5.3^a^ (0.6, 10.0)	−0.6 (− 2.3, 1.2)	0.9 (− 1.0, 2.6)	− 0.4 (− 2.2, 1.4)	0.8 (− 1.1, 2.3)	−1.0 (− 2.8, 0.6)	−0.1 (− 1.9, 1.6)
PSI	1.9 (− 1.7, 5.6)	−1.2 (− 6.2, 3.1)	0.1 (− 1.6, 1.6)	− 0.1 (− 1.6, 1.6)	−0.3 (− 2.1, 1.5)	−0.6 (− 2.3, 1.2)	−0.6 (− 2.2, 1.1)	−0.2 (− 1.9, 1.6)
PRI	− 0.4 (− 4.1, 3.3)	1.3 (− 3.3, 6.4)	−0.2 (− 1.9, 1.5)	0.9 (− 0.7, 2.6)	−1.4 (− 3.3, 0.5)	0.7 (− 1.1, 2.5)	−0.7 (− 2.3, 1.1)	0.1 (− 1.7, 1.8)
VCI	2.4 (− 1.6, 6.5)	7.1^a^ (1.4, 12.7)	−1.5 (− 3.3, 0.2)	0.7 (− 1.2, 2.6)	0.3 (− 1.6, 2.2)	1.4 (− 0.4, 3.3)	−1.2 (− 2.9, 0.8)	1.0 (− 0.7, 3.0)
CANTAB								
Between errors	3.0 (−1.1, 7.1)	− 1.1 (− 6.5, 4.2)	−0.5 (− 2.1, 1.4)	2.2^a^ (0.3, 3.9)	0.3 (− 1.5, 2.2)	−1.6 (− 3.4, 0.4)	−0.1 (− 2, 1.6)	−0.6 (− 2.5, 1.2)
Proportion of successful stops	0.02 (− 0.01, 0.05)	− 0.001 (− 0.04, 0.04)	−0.01^.^ (− 0.02, 0.001)	−0.01 (− 0.02, 0.002)	0.002 (− 0.01, 0.02)	−0.003 (− 0.02, 0.01)	0.01 (− 0.002, 0.02)	0.01 (− 0.001, 0.03)
Stop signal reaction time	−0.05 (− 0.1, 0.02)	−0.03 (− 0.1, 0.1)	0.05^a^ (0.02, 0.1)	0.02 (− 0.02, 0.04)	0.03 (− 0.003, 0.1)	−0.02 (− 0.05, 0.01)	−0.003 (− 0.03, 0.03)	−0.01 (− 0.04, 0.02)

Notes: Linear mixed effect model β coefficients and 95% confidence intervals for SES and all biomarkers for the association with IQ and its sub-scores. All the models were adjusted for age, sex, maternal SPM, HOME total score, and distance from the point source

*SES* Socio-economic status, *UAs* Urinary arsenic, *UCd* Urinary cadmium, *UHg* Urinary mercury, *HHg* Hair mercury, *BPb* Blood lead, *HMn* Hair manganese, *BSe* Blood selenium, *WISC-IV* Wechsler Intelligence Scale for Children edition IV, *IQ* Intellectual Quotient, *VCI* Verbal Comprehension Index, *PRI* Visual-Perceptual Reasoning Index, *WMI* Working Memory Index, *PSI* Processing Speed Index, *GAI* General Ability Index, *CPI* Cognitive Proficiency Index, *CANTAB* Cambridge Neuropsychological Test Automated Battery

^.^ Nearly significant difference from 0

^a^Statistically significant difference from 0

In the mixed effect models the distance from the exposure source showed a positive significant association with the IQ total score and the IQ sub-scores WMI, PRI and VCI. The HOME total score was positively associated with the IQ, GAI and CPI. Maternal SPM was positively associated with the IQ and VCI (data not shown).

Considering the CANTAB scores, we noticed additional associations between executive functions and some of the biomarkers. After adjusting for all the covariates, HMn and BPb was associated with higher number of errors in SWM and higher reaction time in SST. Although not significant, a negative association was also observed between BPb and proportion of successful stops at SST. The SES and the distance from the point source were not found to be associated with the CANTAB scores.

Further analysis was performed adding in the model the interaction term between the SES and each biomarker, and adjusting for all the covariates age, sex, maternal SPM, HOME total score, and distance from the point source. The relationship between the SES and the neurocognitive score remained similar, while we observed different relations between the BPb concentration and the IQ scores, depending on the SES levels: BPb showed a negative significant association with WMI in the lowest SES level (Table 6).

**Table 6:** Linear mixed effect model testing SES and biomarkers interaction term association with neurocognitive outcomes

	SES Mid vs Low	SES High vs Low	log(BPb)	SES Mid vs Low X log(BPb)	SES High vs Low X log(BPb)
IQ	1.0 (−2.8, 4.9)	5.2^a^ (0.3, 10.4)	−2.7 (−5.6, 0.2)	2.2 (− 1.7, 5.8)	1.8 (− 2.8, 6.4)
GAI	1.1 (− 2.9, 5.6)	6.3^a^ (1.0, 11.8)	− 3.5^a^ (− 6.7, − 0.3)	2.8 (− 1.1, 7.3)	2.5 (− 2.0, 7.3)
CPI	0.3 (− 3.6, 4.3)	2.0 (− 2.9, 7.1)	−3.5^a^ (− 6.7, − 0.7)	4.1^a^ (0.2, 7.9)	1.6 (−2.9, 6.0)
WMI	0.9 (− 3.0, 4.9)	6.1^a^ (1.3, 11.0)	− 4.0^a^ (− 6.9, − 1.1)	4.5^a^ (0.8, 8.3)	4.9^a^ (0.4, 9.4)
PSI	2.0 (−2.0, 5.9)	− 0.5 (− 5.9, 4.1)	− 0.8 (− 3.9, 2.3)	1.5 (− 2.8, 5.3)	0.6 (−3.6, 5.2)
PRI	0.01 (− 4.0, 3.7)	2.1 (− 2.8, 7.4)	− 1.3 (− 4.3, 1.8)	0.4 (− 3.4, 4.2)	2.2 (−2.3, 7.0)
VCI	2.4 (−1.8, 6.7)	8.2^a^ (1.9, 14.4)	−2.2 (− 5.3, 0.9)	− 0.2 (− 4.3, 4.0)	2.7 (− 2.5, 7.9)

Notes: Linear mixed effect model β coefficients and 95% confidence intervals (CI) for SES, BPb concentration and the interaction term between SES and BPb for the association with IQ and its sub-scores. All the models were adjusted for age, sex, maternal SPM, HOME total score, and distance from the point source

*SES* Socio-economic status, *BPb* blood lead, *IQ* Intellectual Quotient, *VCI* Verbal Comprehension Index, *PRI* Visual-Perceptual Reasoning Index, *WMI* Working Memory Index, *PSI* Processing Speed Index, *GAI* General Ability Index, *CPI* Cognitive Proficiency Index

^a^Statistically significant difference from 0

A sensitivity analysis was performed to test the influence of the extreme metal concentrations and no differences were found among the statistically significant associations between metal concentrations and the outcomes.

**Fig. 2:**
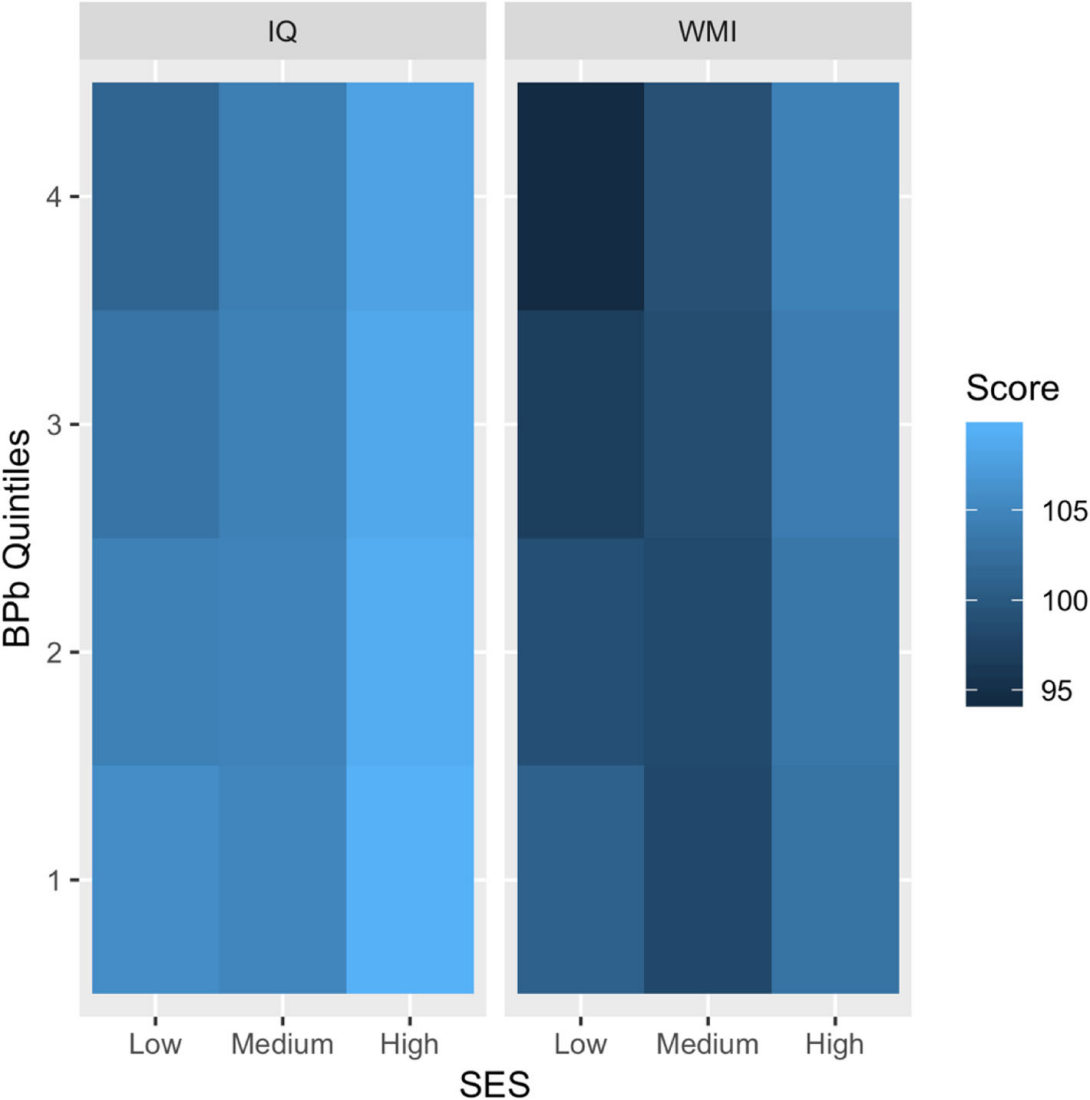
SES and BPb interaction effect on IQ and WMI. Heatmaps of IQ and WMI scores by SES and BPb quintiles, fixing all the other variables (age, maternal SPM, HOME total score, and distance from the point source) equal to their mean and looking at females (males have similar results)

To provide a better understanding of the role of the interaction between SES and BPb on cognitive outcomes, Fig. 2 illustrates the heatmaps for IQ and WMI. For each plot, the predicted values of the model were represented by SES, with BPb concentration varying among the quintiles while the other covariates (age, maternal SPM, HOME total score, and distance from the point source) were fixed at their mean values, and choosing sex equal to female (results for males are similar). Both heatmaps show decreasing levels of the IQ and WMI at increasing quintiles of BPb concentration for the lower level of SES, whereas no variation of the IQ and WMI is notable at medium and high SES levels.

## Discussion

This study revealed that subjects with higher SES showed better neurocognitive scores compared to those with lower SES. A significant interaction of metal exposure with SES was also observed: a higher BPb level for subjects in the lower SES was associated with a statistically significant lower WMI.

The exposure was measured through the biomonitoring measurement of metals known for their neurotoxic properties, and the results showed increasing levels at lower distance from the point source for most metals. To interpret the biomonitoring data, we considered results from other available references. Urinary As resulted higher than the median of 4.5 ng/mL measured by the German Environmental Survey (GerES IV) in 1,734 children, also considering fish eaters (6.2 ng/mL) [51]. It is also higher than the US National Health and Nutrition Examination Survey (NHANES) that showed a median value of 4.8 ng/mL for the 6–11 years age group in the 2013–14 survey [52] and compared to both non-rice eaters (5.3 ng/mL) and rice eaters (8.6 ng/mL) of the same age class [53]. Speciation analysis of As was conducted on 21 urine samples exceeding the value of 50 ng/mL, which is the German biological tolerance value (BAT) for As [54]. The analysis (data not shown) identified six species: As^+ 3^, As^+ 5^, As-Choline, mono-methylaronic acid (MMA), dimethyl-arsenic acid (DMA), and mostly As-Betaine, which is introduced in human body by seafood consumption [55]. The total concentration of the most toxic species As^+ 3^, As^+ 5^ was about 1 ng/mL, which is below the level of potential clinical health hazards.

Urinary Cd was overall (0.4 ng/mL) higher than the median from the NHANES for the 6–11 age group, which was 0.06 ng/mL in the 2009–10 survey [56] and below limit of detection (<LOD) (0.036 ng/mL) in the 2013–14 survey [52], and to the GerES IV (0.08 ng/mL) [51]. A total of 174 (42%) measurements exceeded the human biomonitoring values (HBM-I) of 0.5 ng/mL [57]. The overall median of 477 ng/g for HHg was higher than the values reported from other studies in Brazil (70 ng/g) [58], Czech Republic (190 ng/g) [59]; and in 17 EU Countries, where a geometric mean (GM) of 150 ng/g was measured in a 2010–2012 survey on 120 children 6–11 years [60]. A similar concentration was reported in South West Spain (GM: 407 ng/g, 220 children 6–9 years) [61], France (GM: 370 ng/g, 1364 children 3–17 years [62] and China (GM: 569.8 ng/g, 581 children 0–6-years) [63]. Our values are instead lower compared to those observed in Japan in 335 children of 7 years [64]. Median BPb (8.4 ng/mL) was lower than the median of other Italian studies: 15 ng/mL, measured by our group in a cohort of 11–14 years old children residing in Brescia [16], but similar (9.1 ng/mL) to the PROBE-Adolescent cohort from Latium [65]. It was also lower compared to the GerES IV median of 16.9 ng/mL [51], and higher than the NHANES median value of 7.0 measured in the 2013–14 survey [52]. The median Hair Mn (HMn) value of 135 ng/g measured in this study is higher than the median value of 73 ng/g, obtained from adolescents residing in Brescia after a rigorous preanalytical cleaning methodology [66]. The median of blood Se (BSe) (142 ng/mL) was lower than the NHANES value for the 2013–14 survey of 180 ng/mL.

The SES and maternal educational levels resulted also significantly lower at closer distance from the point source compared to the other study areas; especially when looking at 0–1 km distance we found a striking lower percentage of subjects with high SES. For instance, SES was positively associated with the IQ sub-scores VCI and WMI, which may reflect a specific socio-economic impact on these two cognitive abilities, whereas the PSI sub-score resulted affected by maternal SPM, potentially reflecting a genetic influence.

Significant associations resulted between some of the CANTAB scores and BPb and HMn levels, with a lower proportion of successful stops and higher stop signal reaction time in the SST tests at increasing BPb levels, and higher between errors in the SWM tests at increasing HMn levels. The CANTAB tests are designed to target more specific executive functions, whereas the WISC-IV examines more general composite scores of neuro-cognitive abilities. This may explain why associations with the exposure biomarkers were more evident for the CANTAB tests than for the IQ total and sub-scores, for which the effect of metal exposure may be mitigated by other factors like verbal intelligence or perceptual reasoning.

In fact, by adding an interaction term between the SES and the exposure biomarkers we were able to observe that BPb had a significant negative effect on IQ and WMI for subjects at the lowest SES level. Different studies have highlighted the neurotoxic effects of Pb [8, 67–72], but not assessing the interaction with the SES to test a possible enhancement of the effect. Through the interaction term we were able to find an association between the concentration of BPb and the cognitive abilities among the subjects with a lower SES level that was masked by the higher levels of SES when analyzing the association without including the interaction term in the model.

Due to the cross-sectional nature of the design, it is not possible to infer a causative role of neurotoxic agents on cognitive alterations, and considering the lack of environmental monitoring data, it is not possible to identify the exact exposure sources. Nevertheless, this first assessment yielded relevant initial results although with some limitations that can be addressed in future studies: i) the study power was probably insufficient to fully capture the association between the biomarkers and the IQ scores, therefore a study extension to a larger population is warranted; ii) the exposure assessment lacked environmental measurement and also assessment of organic compounds such as PCBs and dioxins that are also known for neurodevelopmental effects; iii) earlier more vulnerable exposure windows including prenatal and postnatal periods, which were not assessed by the biomonitoring assessment that covered only current exposure.

Moreover, future additional analysis will be needed to address the potential interactions between the different metals within the context of mixed exposure. Finally, the socio-economic factors independently responsible for the impact on the children’s IQ, need to be further investigated and more accurately defined in view of future potential preventive intervention.

## Conclusions

This study provides evidence of neurological effects potentially due to the environmental exposure to metal exposure and their interaction with socio-economical stressors in the area of Taranto, Italy. To the best of our knowledge, this is the first study investigating the impact of metal exposure and socio-economical stressors on neurocognitive outcomes among school age children living in this highly polluted environment of southern Italy.

## Data Availability

The datasets used and/or analyzed during the current study are available from the corresponding author on reasonable request.

## Abbreviations

ADHD: Attention Deficit Hyperactivity Disorder
As: Arsenic
BPb: Blood lead
BSe: Blood selenium
CANTAB: Cambridge Neuropsychological Test Automated Battery
CBCL: Child Behavior Checklist
Cd: Cadmium
CPI: Cognitive Proficiency Index
GAI: General Ability Index
Hg: Mercury
HHg: Hair mercury
HMn: Hair manganese
HOME: Home Observation for Measurement of the Environment
IQ: Intellectual Quotient
LOD: Limit of detection
Mn: Manganese
Pb: Lead
PRI: Visual-Perceptual Reasoning Index
PSI: Processing Speed Index
RVP: Rapid Visual Information Processing
SES: Socio-economic status
SOC: Stockings of Cambridge
SPM: Standard Progressive Matrices
SST: Stop Signal Task
SWM: Spatial Working Memory
UAs: Urinary arsenic
UCd: Urinary cadmium
UHg: Urinary mercury
VCI: Verbal Comprehension Index
WISC-IV: Wechsler Intelligence Scale for Children edition IV
WMI: Working Memory Index
